# The AMP–antibiotic–microbiota triad in IBD: a mechanistic framework for dysregulated antimicrobial defense

**DOI:** 10.3389/fimmu.2026.1779550

**Published:** 2026-03-17

**Authors:** Yuyuan Hu, Yan Li, Qiang Zhang, Qiaobo Tan, Haoze Liu, Yuhang Yang, Chenfei Jin, Wei Zhang, Jinghan Jia, Jinxi Wang

**Affiliations:** 1 Division of Colorectal Surgery, Third Hospital of Shanxi Medical University, Shanxi Bethune Hospital, Shanxi Academy of Medical Sciences, Tongji Shanxi Hospital, Taiyuan, China; 2 Neurology, Shanxi Bethune Hospital, Shanxi Academy of Medical Sciences, Third Hospital of Shanxi Medical University, Tongji Shanxi Hospital, Taiyuan, China; 3 Urology Department, Third Hospital of Shanxi Medical University, Shanxi Bethune Hospital, Shanxi Academy of Medical Sciences, Tongji Shanxi Hospital, Taiyuan, China

**Keywords:** antibiotics, antimicrobial imbalance hypothesis, antimicrobial peptides (AMPs), dysbiosis, gut microbiota, host–microbe interaction, inflammatory bowel disease (IBD), paneth cells

## Abstract

Inflammatory bowel disease (IBD) represents a chronic relapsing disorder driven by a loss of homeostatic balance between the host immune system and the intestinal microbiota. Endogenous antimicrobial peptides (AMPs), produced primarily by epithelial and immune cells, function in concert with commensal microorganisms to preserve mucosal integrity and barrier function. Disruption of this antimicrobial equilibrium—through genetic susceptibility such as NOD2 mutations or environmental perturbations including antibiotic overuse—can impair antimicrobial defense, distort microbial composition, and initiate chronic inflammation. Recent investigations have revealed distinct alterations in AMP expression across IBD subtypes. In Crohn’s disease, Paneth cell–derived α-defensins (HD5 and HD6) are markedly diminished in the ileal mucosa, whereas colonic, segmental IBD exhibits inadequate induction of β-defensins and LL-37. Conversely, in actively inflamed regions, certain AMPs such as human β-defensin-2 (HBD2) and lysozyme are strongly upregulated, reflecting a compensatory response to inflammatory cell infiltration and microbial invasion. Beyond host-derived peptides, broad-spectrum antibiotic exposure profoundly reshapes commensal communities, attenuates basal pattern-recognition receptor signaling, and secondarily perturbs AMP regulation—creating a feedback loop that amplifies dysbiosis. Here, we conceptualize these interactions as an integrated AMP–antibiotic–microbiota triad, in which endogenous antimicrobial regulation, exogenous antimicrobial pressure, and microbial ecological resilience dynamically co-determine mucosal stability. By positioning AMPs within this tripartite regulatory framework, this review delineates how antimicrobial imbalance arises across IBD subtypes, compares emerging therapeutic strategies—including AMP enhancement, microbiota-sparing antibiotic regimens, fecal microbiota transplantation, and metabolite-guided interventions—and highlights implications for precision recalibration of antimicrobial homeostasis in IBD.

## 1 Introduction

Inflammatory bowel disease (IBD), encompassing Crohn’s disease and ulcerative colitis, is a multifactorial disorder arising from genetic susceptibility combined with disruption of host–microbiota homeostasis (1). Under physiological conditions, the intestine maintains a finely tuned “antimicrobial balance, “ in which host-derived antimicrobial peptides and barrier mechanisms shape microbial communities by supporting beneficial species while restraining potential pathogens (2). This equilibrium is further modulated by environmental factors such as diet, microbial exposure, and lifestyle (3). When genetic predisposition or environmental perturbations disturb this balance, maladaptive host–microbe interactions ensue, driving chronic immune activation and mucosal injury characteristic of IBD (4).

Consistent with this concept, patients with IBD exhibit a characteristic dysbiosis marked by depletion of beneficial anaerobes—including short-chain fatty acid–producing Bacteroidetes and Firmicutes—and expansion of facultative anaerobes such as Escherichia coli and Enterobacteriaceae (5), a shift increasingly recognized as a contributor to intestinal inflammation. In parallel, more than 200 IBD-associated genetic loci have been identified, many of which regulate epithelial barrier function and microbial sensing (6). Variants in key pathways, including NOD2, ATG16L1, and XIAP, impair epithelial antimicrobial defense and weaken host control over commensal microbes, thereby predisposing susceptible individuals to microbially driven intestinal inflammation (7–9). Environmental influences further compound this risk: large-scale cohort studies show that childhood exposure to antibiotics—particularly broad-spectrum and repeated courses—nearly doubles the likelihood of developing IBD later in life (10, 11). Conversely, early-life exposure to domestic or farm animals has been associated with a reduced risk of IBD, supporting a protective role for microbial diversity during immune development (12).

Collectively, these findings indicate that IBD arises from a mismatch between intrinsic antimicrobial defenses and extrinsic environmental pressures, whereby insufficient defense permits microbial overgrowth, while excessive antimicrobial activity disrupts symbiotic homeostasis. Building on this framework, this review outlines a mechanistic continuum from antimicrobial balance to imbalance, inflammatory injury, and restorative intervention, focusing on interactions among antimicrobial peptides, antibiotics, and the gut microbiota.

## 2 Literature search strategy

A structured literature search was conducted to identify relevant studies addressing antimicrobial peptides (AMPs), antibiotic exposure, and microbiota–host interactions in inflammatory bowel disease (IBD). Electronic databases including PubMed, Web of Science, and Scopus were searched for articles published from January 2000 to March 2025.

Search terms included combinations of the following keywords: “inflammatory bowel disease, “ “Crohn’s disease, “ “ulcerative colitis, “ “antimicrobial peptides, “ “defensins, “ “LL-37, “ “RegIII, “ “Paneth cells, “ “antibiotics, “ “microbiota, “ “dysbiosis, “ “IL-22, “ “STAT3, “ and “host–microbe interaction.”

Original research articles, translational studies, and high-quality review articles written in English were considered. Studies focusing exclusively on non-intestinal systems or unrelated infectious contexts were excluded. Emphasis was placed on mechanistic investigations, ecological analyses, and clinically relevant translational data to support the integrative framework proposed in this review.

## 3 Endogenous antimicrobial peptides: intelligent guardians of the mucosal barrier

Antimicrobial peptides (AMPs) serve as the front-line sentinels of innate immunity within the intestinal mucosa. Secreted by epithelial and immune cells, these small cationic peptides can directly kill microorganisms while also modulating mucosal immune responses (13). The principal intestinal AMP families include Paneth cell–derived α-defensins (HD5 and HD6); β-defensins (HBD1–4) produced by epithelial and immune cells; cathelicidins such as LL-37 secreted by neutrophils and epithelial cells; C-type lectins represented by the RegIII family; and S100 calcium-binding proteins (for example, calprotectin), together with lysozyme and lactoferrin (14–17). Acting in concert, these peptides form a dynamic chemical barrier over the intestinal mucosa that preserves microbial symbiosis while preventing pathogen invasion. AMPs safeguard intestinal barrier integrity through multiple complementary mechanisms. Cationic peptides such as defensins and LL-37 can bind to bacterial membranes and form pores, leading to direct microbial lysis (16). C-type lectins of the RegIII family recognize bacterial peptidoglycan and spatially restrict bacterial proximity to the epithelium by modulating mucus organization—thus maintaining a crucial “spatial segregation” between host and microbes (18). In addition, certain AMPs, including cathelicidins, function as chemotactic and immunomodulatory molecules; for example, human β-defensins (HBD1 and HBD2) recruit CCR6-expressing immune cells, such as dendritic cells and T cells, to modulate mucosal immune responses (19).

### 3.1 Mechanisms and regulatory pathways governing antimicrobial peptide activity

**Figure 1:**
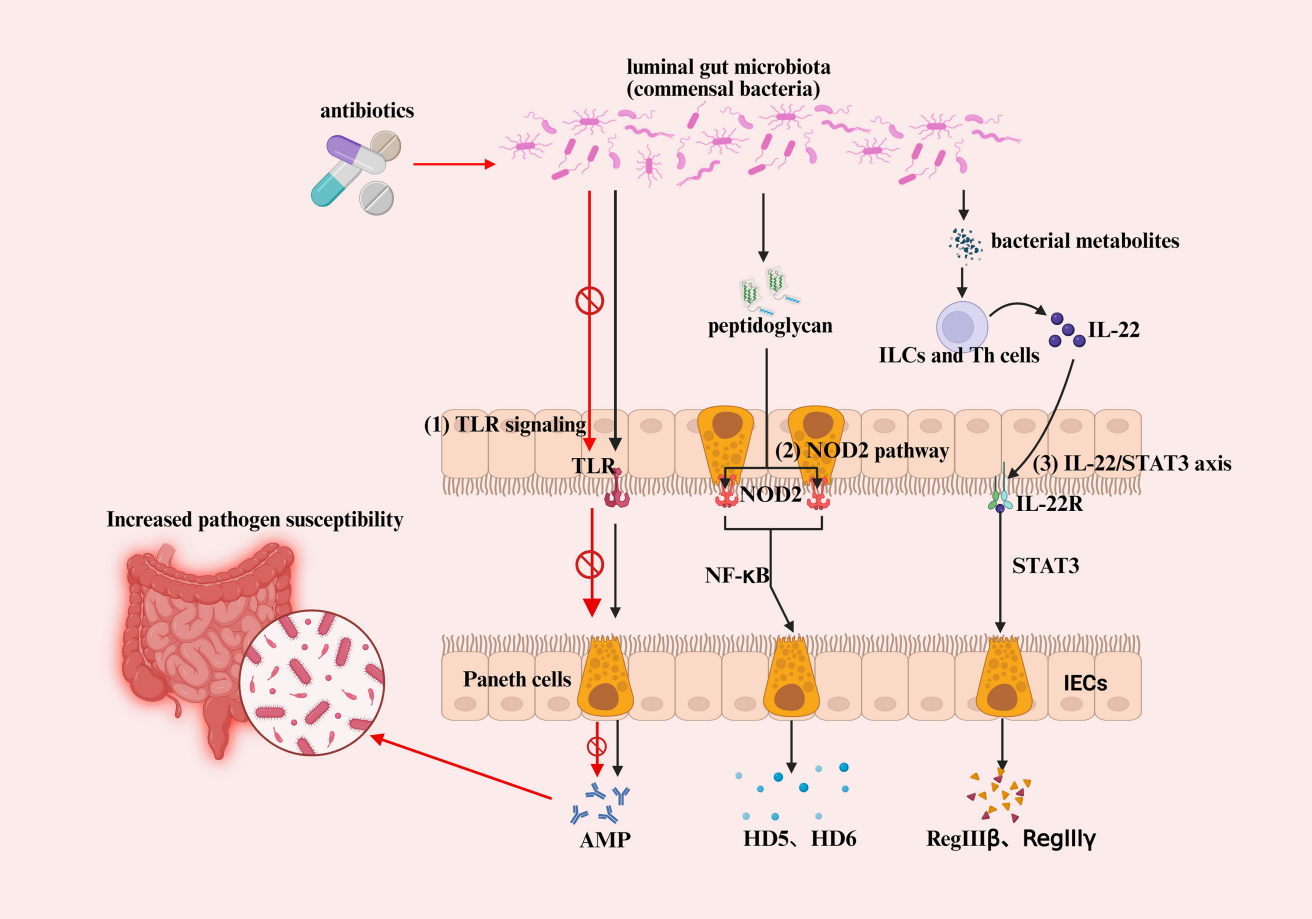
This schematic illustrates how commensal-derived signals, innate immune receptors, and cytokine networks coordinate epithelial antimicrobial peptide (AMP) production and how antibiotic disruption of these pathways increases pathogen susceptibility. (1) TLR signaling: Commensal bacteria in the intestinal lumen activate epithelial and Paneth-cell Toll-like receptors (TLRs), promoting the expression of RegIII family AMPs. Loss of microbial stimulation after broad-spectrum antibiotics suppresses TLR pathways and reduces basal AMP levels, thereby increasing pathogen susceptibility. (2) NOD2 pathway: Paneth-cell NOD2 senses bacterial peptidoglycan and activates NF-κB, inducing α-defensin (HD5, HD6) production. NOD2 thus acts as a key intracellular sensor linking microbial signals to Paneth-cell AMP secretion. (3) IL-22/STAT3 axis: IL-22 released by innate lymphoid cells (ILCs) and T helper cells—stimulated by bacterial metabolites such as tryptophan-derived AhR ligands and short-chain fatty acids—binds to IL-22R on epithelial cells and activates STAT3, leading to robust induction of RegIIIβ, RegIIIγ, and other epithelial AMPs.Collectively, these three pathways form a microbiota–immune–epithelial regulatory axis, ensuring appropriate AMP production to maintain mucosal barrier integrity while preventing overgrowth of pathogenic bacteria.

Antimicrobial peptide expression in the intestinal mucosa is regulated by an integrated network of microbial sensing and cytokine-driven pathways that calibrate epithelial defense. As summarized in **Figure 1**, this regulation is coordinated by three principal axes: TLR-mediated sensing of commensal-derived signals, NOD2-dependent recognition of bacterial peptidoglycan driving Paneth cell α-defensin secretion, and IL-22–STAT3 signaling inducing RegIII lectins and other inducible AMPs. Together, these pathways maintain basal antimicrobial tone and spatial segregation between the microbiota and the epithelium. Notably, the NOD2–α-defensin axis represents the most clearly defined mechanistic link between microbial recognition and Paneth cell dysfunction in ileal Crohn’s disease and is discussed in detail in Section 4.1.1.

#### 3.1.1 Pattern recognition receptors

Toll-like receptors (TLRs) are membrane-bound pattern-recognition receptors that regulate the abundance of commensal microorganisms and help maintain tissue integrity in mammals (20). On intestinal epithelial and Paneth cells, TLRs sense microbial signals within the gut lumen and induce the expression of antimicrobial peptides such as RegIII (21). The expression of RegIIIγ in epithelial cells requires TLR stimulation, and commensal bacteria, through continuous activation of TLR signaling, sustain the basal production of antimicrobial peptides by Paneth cells—an effect thought to protect against pathogen colonization (22). Conversely, when broad-spectrum antibiotics eliminate commensal microbial stimuli, these TLR pathways become effectively “silenced, “ leading to a reduction in AMP production and, unintentionally, an increased susceptibility to pathogenic invasion (23).

#### 3.1.2 NOD-like receptors

Intestinal bacteria and the host interact through Toll-like receptors (TLRs) and NOD-like receptors (NLRs). Nucleotide-binding oligomerization domain–containing protein 2 (NOD2) is an intracellular receptor specifically expressed in Paneth cells of the small intestine. It recognizes bacterial peptidoglycan fragments and activates the NF-κB signaling pathway, thereby promoting the secretion of the α-defensins HD5 and HD6 by Paneth cells (24, 25). Under physiological conditions, NOD2 signaling promotes α-defensin gene transcription and granule exocytosis, positioning NOD2 as a central link between microbial sensing and antimicrobial peptide production.

#### 3.1.3 Cytokine pathways

Interleukin-22 (IL-22), secreted primarily by innate lymphoid cells and helper T cells, binds to epithelial IL-22R receptors and activates the STAT3 signaling cascade—an essential mechanism driving antimicrobial peptide (AMP) induction in the intestinal mucosa (26). Experimental studies have shown that IL-22/STAT3 signaling markedly upregulates the expression of RegIIIβ, RegIIIγ, and members of the S100 antimicrobial protein family, thereby strengthening mucosal barrier defenses and reducing bacterial translocation (27). IL-22 itself is produced by intestinal ILC3 cells in response to microbial metabolites such as tryptophan-derived aryl hydrocarbon receptor (AhR) ligands and short-chain fatty acids, forming a key node within the “microbiota–immune–epithelial” regulatory axis (28, 29). For instance, indole derivatives generated by commensal bacteria can activate AhR signaling in ILC3s, enhancing IL-22 secretion and subsequently promoting epithelial defensin production—illustrating how the microbiota actively shapes its own ecological balance through host antimicrobial regulation (30).

In summary, intestinal AMP expression is governed by an integrated network involving TLR-, NOD2-, and IL-22–mediated signaling, which translates microbial cues into epithelial defense responses to maintain mucosal balance. Disruption of this coordinated regulation can disturb antimicrobial homeostasis—laying the groundwork for the altered AMP patterns observed in inflammatory bowel disease.

Importantly, this tightly coordinated signaling network is highly sensitive to ecological perturbations imposed by antibiotic exposure (31, 32). The IL-22–STAT3 axis is tightly coupled to metabolite-producing commensal taxa, and class-dependent antibiotic perturbation can disrupt this metabolite–immune interface (33, 34). Such ecological reprogramming attenuates downstream AMP induction and links exogenous antimicrobial pressure to endogenous mucosal defense regulation (33).

Under physiological conditions, these interconnected pathways maintain AMP homeostasis (35). In IBD, disruption of this regulatory network manifests as compartment-specific alterations in AMP expression, providing a mechanistic basis for the heterogeneous AMP phenotypes observed in ulcerative colitis and Crohn’s disease (36, 37).

### 3.2 Bidirectional alterations of antimicrobial peptides in inflammatory bowel disease and their clinical implications

In inflammatory bowel disease (IBD), antimicrobial peptide (AMP) expression is characterized by a bidirectional imbalance, combining impaired baseline antimicrobial defense with compensatory overactivation of inducible peptides. This loss–overcompensation dynamic reflects disrupted host–microbiota equilibrium and contributes to IBD pathogenesis.

#### 3.2.1 Disruption of the paneth cell–microbiota interaction and loss of antimicrobial equilibrium

In patients with ileal Crohn’s disease, Paneth cell–derived α-defensins (HD5 and HD6) are markedly reduced. Wehkamp and colleagues demonstrated that patients carrying NOD2 mutations exhibit significantly lower ileal HD5 and HD6 mRNA levels than those with the wild-type allele, indicating that impaired NOD2 signaling compromises α-defensin expression (37). NOD2 is predominantly expressed in Paneth cells, where it functions as a microbial sensor linking bacterial recognition to defensin secretion. Disruption of this pathway results in insufficient defensin release, leading to microbial imbalance, persistent mucosal stimulation, and chronic intestinal inflammation characteristic of Crohn’s disease (38, 39). Moreover, chronic mucosal inflammation further impairs Paneth cell secretory function, reducing α-defensin output, weakening epithelial antimicrobial barriers, and facilitating bacterial translocation that perpetuates inflammation (40).

Moreover, several monogenic disorders provide more direct evidence linking innate immune defects to intestinal inflammation. Notably, private variants in the X-linked inhibitor of apoptosis protein (XIAP) have been identified in approximately 4% of male pediatric patients with inflammatory bowel disease (40). Worthey et al. first characterized XIAP deficiency as an underlying primary disorder in a subset of IBD patients, highlighting its pivotal role in maintaining mucosal immune homeostasis (41). Strigil et al. demonstrated that XIAP deficiency disrupts Paneth cell homeostasis, rendering the cells highly susceptible to microbiota-derived stimuli, TNF, and RIPK1/RIPK3-mediated cell death. Loss of Paneth cell–derived antimicrobial peptides in XIAP^-^/^-^ mice resulted in marked microbiota alterations and increased susceptibility to microbially driven, granulomatous ileitis—an outcome that could be prevented by restoring antimicrobial peptide expression (8). Together, these findings highlight Paneth cell dysfunction as a genetic nexus between impaired antimicrobial defenses and intestinal inflammation and illustrate a broader, bidirectional imbalance of antimicrobial peptides across the IBD spectrum. **Table 1** summarizes the distinct AMP expression profiles characteristic of Crohn’s disease and ulcerative colitis.

**Table 1:** Antimicrobial peptide imbalance as a mechanistic determinant in Crohn’s disease versus ulcerative colitis.

Antimicrobial peptide	Expression in Crohn’s disease	Expression in ulcerative colitis	Functional significance	Reference
HD5 Paneth cell peptide	↓ Reduced in ileal CD. Notably low in patients with NOD2 risk variants	No major deficiency.	Major Paneth cell defensin with broad bactericidal activity (kills bacteria, shapes gut microbiota). Protects mucosal integrity; deficiency permits microbial dysbiosis and invasion.	(37, 123)
HD6 Paneth cell peptide	↓ Reduced in ileal CD. Diminished Paneth cell HD6 impairs mucosal defense nets.	Not normally expressed in colon. No inherent deficit reported in UC.	Paneth cell defensin forming non-cytolytic nanonets that trap microbes. Complements HD5’s action; loss of HD6 leaves mucosa prone to bacterial penetration.	(82, 124)
HBD1 Epithelial constitutive defensin	↓ Decreased baseline expression in CD colonic mucosa. In inflamed CD, activation is limited, and low thioredoxin in Crohn’s lesions may reduce hBD-1’s antimicrobial activation.	↓ Decreased (marginally) in UC inflamed mucosa as well. Like in CD, hBD-1 is constitutively made and not upregulated by inflammation. Any decrease mainly reflects epithelial injury rather than specific UC defect.	Constitutive defensin active against gut microbes. Helps maintain microbiota homeostasis; diminished levels weaken baseline mucosal defense.	(36, 42, 125)
HBD2 Epithelial inducible defensin	Minimal induction in CD – fails to upregulate even during inflammation. Colonic CD shows impaired HBD2 induction, contributing to deficient barrier defense.	↑ Strongly elevated in active UC mucosa. HBD2 is barely present in healthy colon but markedly upregulates with inflammation in UC.	Potent broad-spectrum defensin; inducible via NF-κB/AP-1 pathways in response to infection/inflammation irjournal.org. High HBD2 in UC helps limit bacteria during flares, whereas lack of HBD2 induction in CD leaves the mucosa vulnerable.	(36, 52)
HBD3 Epithelial inducible defensin	No strong induction in CD. Any upregulation is muted compared to UC, partly contributing to weaker antimicrobial shield in CD.	↑ Elevated in active UC. Induced alongside HBD2 during flares. HBD3 levels in UC > CD under similar inflammatory conditions.	Broad-spectrum cationic defensin; enhances barrier defense. Inducible by microbes/cytokines; provides robust antimicrobial activity in UC flares. Limited HBD3 in CD implies a gap in inducible defense.	(36, 126, 127)
LL-37 Neutrophils, macrophages, epithelial cells	No significant change in colonic CD.	↑ Increased in UC colonic mucosa. Intestinal LL-37 expression is upregulated by microbiota metabolites and vitamin D; UC shows higher levels in inflamed tissue.	Broad-spectrum antimicrobial peptide and immunomodulator. Crucial for mucosal healing: cathelicidin-deficient mice suffer more severe colitis, highlighting its protective role in UC.	(128, 129)
Calprotectin Neutrophil cytosolic protein	↑ High in active CD. Levels correlate with disease activity, though purely small-bowel CD can produce lower fecal calprotectin than colonic disease.	↑ High in active UC. Often markedly elevated due to extensive mucosal neutrophil infiltration; falls with mucosal healing.	Calcium-binding heterodimer with antimicrobial properties: sequesters Zn/Mn to inhibit bacterial growth. Also serves as a pro-inflammatory mediator and a clinical biomarker for intestinal inflammation.	(130, 131)
Lactoferrin Neutrophil granules, secretory fluids	↑ Elevated in CD. Fecal lactoferrin is often positive in active CD, especially with colonic involvement, indicating neutrophil degranulation in the gut.	↑ Elevated in UC. High fecal lactoferrin in UC reflects substantial neutrophil presence in the colonic mucosa. Levels drop upon effective treatment.	Iron-binding glycoprotein with broad antimicrobial and immunomodulatory effects: chelates iron to starve pathogen, disrupts bacterial membranes, and enhances phagocytosis. Clinical marker of neutrophilic inflammation.	(132–134)
Regenerating family protein RegIIIγ	↑ Upregulated during intestinal inflammation. In CD, Paneth and enteroendocrine cells can increase RegIII (HIP/PAP) expression in inflamed segments. No strong differential vs UC; generally, part of innate response in both.	↑ Upregulated in active UC mucosa. Colonic epithelial cells highly express HIP/PAP (Reg3α) under inflammatory conditions.	C-type lectin that binds peptidoglycan and kills bacteria. Mainly from Paneth cells and colon endocrine cells, it is strongly induced by microbes via TLR-MyD88 signaling. Helps maintain spatial segregation of microbiota; increased production in IBD is thought to bolster the impaired mucus barrier.	(18, 35, 83, 135)
Elafin Epithelial antiprotease	↓ Decreased in active CD. Mucosal elafin levels in inflamed colonic tissue are lower than in uninflamed areas or controls. Limited upregulation in CD during flares suggests a blunted protective response.	↓ Decreased in active UC. Elafin expression rises in UC during remission, but drops sharply with active inflammation. Overall, active IBD shows an elafin deficit relative to normal tissue.	Serine protease inhibitor with antimicrobial and anti-inflammatory properties. Protects mucosal tissues from excessive protease-mediated damage. Lower elafin in active IBD is associated with worse inflammation, whereas higher levels are protective. Experimental overexpression of elafin mitigates colitis severity in mice.	(39, 136, 137)
SLPI Mucosal antiprotease	↑ Increased in CD inflamed mucosa. Intestinal SLPI expression is inducible by inflammatory stimuli; CD patients show higher SLPI in affected segments as a protective countermeasure.	↑ Increased in UC inflamed mucosa. Active UC upregulates SLPI locally (released by epithelial cells and macrophages) in response to elevated neutrophil proteases, reflecting a protective feedback mechanism.	Antiprotease that inhibits neutrophil elastase and other serine proteases, preserving epithelial barrier function. Also possesses direct antimicrobial and tissue-healing effects. Induced during inflammation to prevent excessive tissue injury. SLPI-deficient mice suffer more severe colitis and barrier loss, whereas administering SLPI or other protease inhibitors ameliorates colitis.	(136, 138, 139)

Crohn’s disease exhibits impaired baseline antimicrobial immunity driven by Paneth cell dysfunction and insufficient inducible epithelial AMP responses, whereas ulcerative colitis features a hyper-inducible antimicrobial state characterized by elevated β-defensins, cathelicidin, and neutrophil-derived peptides during active inflammation. These divergent antimicrobial programs reflect fundamentally distinct mucosal immune ecologies and illuminate the mechanistic contribution of AMP imbalance to the host–microbiota interface in IBD.

#### 3.2.2 The altered expression of inducible mucosal antimicrobial peptides

In active IBD, several inducible antimicrobial peptides (AMPs) are markedly upregulated in the intestinal mucosa, serving not only as indicators of inflammation but also as contributors to the ongoing host–microbiota interplay.

##### 3.2.2.1 β-defensin-2

Unlike constitutively expressed HBD-1, β-defensin-2 (HBD2) is minimally expressed in the healthy colon but markedly induced in inflamed IBD mucosa. This induction is particularly pronounced in ulcerative colitis (UC), where HBD2 expression exceeds that observed in Crohn’s disease (CD); HBD2 mRNA is detectable in approximately 53% of UC biopsies compared with 34% in CD, indicating stronger innate immune activation in UC (42). Excessive HBD2 induction is considered a mucosal response to heightened microbial stimulation and parallels the expansion of opportunistic pathobionts, reflecting a stress-driven epithelial attempt to limit bacterial overgrowth (43).

##### 3.2.2.2 Cathelicidin (LL-37)

The expression pattern of cathelicidin (LL-37) in IBD is also heterogeneous. Several studies report a marked increase in LL-37 within inflamed mucosa in both UC and Crohn’s disease, whereas others describe a more selective upregulation in UC with little or no change in a subset of Crohn’s disease patients (44, 45). Overall, LL-37 appears to function as part of the mucosal innate defense system and is likely released in larger amounts during active inflammation, where it may contribute to antimicrobial protection and tissue repair (44). Interestingly, circulating and tissue LL-37 levels also seem to track with disease activity: higher concentrations are often observed during remission, while some patients in the active phase show a relative depletion, supporting the notion that LL-37 exerts a protective, rather than pathogenic, role (46).

##### 3.2.2.3 Calprotectin and lactoferrin

Calprotectin and lactoferrin, by contrast, are antimicrobial proteins released predominantly by neutrophils that accumulate in large numbers within the intestinal lumen during IBD flares. Marked elevations of fecal calprotectin and lactoferrin have become well-established, noninvasive markers of mucosal inflammation, widely used to distinguish inflammatory from functional diarrhea and to assess disease activity (47). Both proteins mirror the degree of innate immune activation at the mucosal surface: calprotectin exerts antimicrobial effects by chelating essential metal ions, whereas lactoferrin restricts bacterial growth through high-affinity iron binding (48). In patients with IBD, fecal concentrations of these proteins correlate closely with endoscopic inflammation and have been shown to predict disease relapse and to monitor therapeutic response (49, 50). Collectively, the upregulation of these AMPs represents a protective effort by the intestinal epithelium to counter microbial perturbations, while also reflecting the broader struggle to re-establish mucosal homeostasis during active disease. However, the extent to which these quantitative shifts restore effective mucosal antimicrobial defense remains unclear. Current evidence suggests that in UC the inducible AMP profile reflects quantitative predominance within conserved peptide classes rather than the emergence of a novel antimicrobial repertoire, with functional activity shaped by the inflammatory microenvironment (51–53).

In summary, antimicrobial peptide (AMP) expression in IBD displays a dual imbalance, combining loss of key antimicrobial defenses—such as Paneth cell α-defensins—with compensatory overexpression of inducible peptides, including HBD2, LL-37, calprotectin, and lactoferrin. Defensin deficiency weakens epithelial integrity and promotes dysbiosis, whereas excessive induction of secondary AMPs reflects amplified inflammation that can further shape microbial composition. This disturbed AMP equilibrium disrupts host–microbiota homeostasis and sustains chronic intestinal inflammation, highlighting restoration of physiologic AMP function as a potential strategy to improve mucosal stability and disease control.

## 4 Antibiotics as a double-edged modifier of gut homeostasis in IBD

Antibiotics, although primarily employed as exogenous antimicrobial agents in the treatment of infections, exert far more complex effects within the gut’s delicate microbial ecosystem than simple pathogen eradication. Their use can profoundly reshape the intestinal microbiota and, in some cases, trigger a cascade of unforeseen ecological consequences, including secondary impacts on the host’s antimicrobial peptide (AMP) system (54).

Broad-spectrum antibiotics can rapidly and markedly reduce the overall bacterial load and diversity of the gut microbiota, leading to a substantial depletion of beneficial commensals and a relative expansion of opportunistic pathogens and fungi, ultimately driving the community toward a more homogeneous configuration (55). For example, following broad-spectrum antibiotic treatment, mice exhibit a pronounced decline in the abundance of Bacteroidetes and Firmicutes, accompanied by an increased proportion of Proteobacteria—including Escherichia coli—resulting in an overall reduction in microbial diversity (54, 56). Notably, the alterations in gut microbiota induced by antibiotics can be long-lasting. In some individuals, the microbial community fails to return to its pre-treatment equilibrium even months after discontinuing antibiotic therapy (57). Such persistent effects have raised concerns about the overuse of antibiotics, particularly given evidence that each antibiotic course during childhood is associated with a substantially increased risk of developing IBD later in life (10, 11). Moreover, the impact of antibiotic exposure is not uniform: different classes of antibiotics vary in their antimicrobial spectrum and mechanisms of action, leading to distinct patterns of microbiota disruption and differential effects on AMP-related pathways.

### 4.1 Ecological reprogramming and class-dependent AMP modulation

Antibiotic exposure reshapes the intestinal microbiota in a class-dependent manner by selectively depleting metabolically active commensal taxa rather than uniformly suppressing microbial abundance (58, 59). Of particular relevance are short-chain fatty acid (SCFA)–producing Clostridium clusters IV and XIVa and indole-generating Bacteroides species, which provide tonic metabolic signals that contribute to mucosal immune homeostasis, including IL-22–STAT3 signaling and antimicrobial peptide (AMP) regulation (34, 60).

Different antibiotic classes are associated with distinct patterns of ecological restructuring within the intestinal microbiota (59). Fluoroquinolones and other broad-spectrum agents selectively deplete anaerobic taxa within Bacteroides and obligate Firmicutes lineages, perturbations linked to reduced availability of indole-derived aryl hydrocarbon receptor ligands and short-chain fatty acids (SCFAs) (34, 61, 62). Nitroimidazoles, which primarily target strict anaerobes, are similarly associated with depletion of butyrate-producing Clostridia, thereby attenuating metabolite-mediated mucosal immune conditioning. Broad-spectrum β-lactams may further diminish functional redundancy within SCFA-generating communities (63). Because IL-22–dependent antimicrobial peptide (AMP) induction is influenced by microbial metabolic cues, such class-dependent ecological perturbations may attenuate basal defensin expression and modulate mucosal antimicrobial tone (33). These mechanistic relationships are summarized in **Table 2**.

**Table 2:** Class-dependent ecological and immunological consequences of antibiotic exposure.

Antibiotic class	Dominant ecological impact	Functional metabolic consequence	Immune signaling node affected	Downstream epithelial effect	Predicted AMP modulation	Reference
Fluoroquinolones	Restructuring of anaerobic Bacteroides and Firmicutes communities	↓ Indole derivatives and SCFA-associated taxa	Reduced AhR engagement and IL-22–dependent signaling	Attenuated epithelial IL-22–STAT3 tone	Reduced inducible defensins and RegIII family lectins	(33, 34, 61)
Nitroimidazoles	Depletion of strict anaerobes, including butyrate-associated Clostridia	↓ Butyrate production	Impaired metabolite-mediated mucosal immune conditioning	Reduced epithelial immune activation programs	Attenuated AMP induction	(33, 57, 63)
Cephalosporins	Broad perturbation of Firmicutes-dominated communities	↓ SCFA diversity and functional redundancy	Reduced tonic mucosal immune signaling	Lower basal IL-22–dependent epithelial activity	Decreased basal AMP expression	(18, 58, 140)
Carbapenems	Multi-lineage anaerobic depletion with severe community contraction	↓ SCFAs and other microbial metabolites	Disrupted metabolite–immune integration	Context-dependent modulation of IL-22–STAT3 axis	Context-dependent AMP dysregulation	(34, 141)

This table summarizes representative patterns of ecological restructuring induced by major antibiotic classes and their putative downstream consequences on microbial metabolite availability, mucosal immune signaling, and antimicrobial peptide (AMP) regulation.

Distinct antibiotic classes selectively perturb metabolite-producing commensal taxa, including short-chain fatty acid (SCFA)–generating Clostridium clusters and indole-producing Bacteroides lineages. These ecological shifts are associated with altered engagement of immune signaling nodes—such as the aryl hydrocarbon receptor (AhR) and IL-22–STAT3 axis—which in turn modulate epithelial AMP output.

The relationships presented reflect integrative interpretation of current experimental and translational evidence and are intended to illustrate mechanistic links within the AMP–antibiotic–microbiota triad rather than imply uniform causal effects across all clinical contexts.

Experimental models support this link between microbial depletion and AMP regulation (35). In antibiotic-treated or germ-free mice, reduced Paneth cell α-defensin expression accompanies loss of microbiota-derived stimulation, underscoring the dependence of basal AMP maintenance on commensal-derived signals (18, 64). Thus, antibiotic-induced dysbiosis should be viewed not only as ecological disturbance, but as a metabolite-dependent immune recalibration that intersects directly with IL-22–STAT3–mediated AMP homeostasis.

### 4.2 From broad-spectrum suppression to selective modulation: evolving antibiotic concepts in IBD therapy

Compared with broadly acting antibiotics, a shift toward narrower or more selective antimicrobials has gained attention as a way to modulate gut microbial communities in IBD. Rifaximin is one such agent; it remains almost entirely within the intestinal lumen and shows activity against a range of Gram-positive, Gram-negative, and anaerobic species (65). Studies suggest that rifaximin can dampen the expansion of harmful Enterobacteriaceae—particularly certain E. coli strains—while leaving some beneficial bacteria relatively unaffected. This adjustment of the microbial environment may reduce endotoxin exposure and contribute modest anti-inflammatory effects (66, 67). Beyond these ecological effects, rifaximin acts as a pregnane X receptor (PXR) agonist in intestinal epithelial cells (68). Epithelial PXR activation suppresses NF-κB–dependent inflammatory signaling and modulates downstream innate immune pathways (69). Through these mechanisms, rifaximin may contribute to the modulation of antimicrobial peptide (AMP) expression, including defensins and RegIIIγ (18). Given its minimal systemic absorption, current evidence supports direct epithelial PXR activation as a major contributor to rifaximin-associated immune modulation, whereas microbiota “eubiotic” shifts likely play a complementary role (65, 70). Notably, in clinical trials involving patients with active Crohn’s disease, rifaximin produced higher rates of clinical improvement than placebo in part of the cohort (71).

Other antibiotics, including metronidazole and ciprofloxacin, continue to be used in Crohn’s disease, especially for perianal complications or to lower the risk of postoperative recurrence (72). Metronidazole primarily targets anaerobes, and short-term therapy can suppress Bacteroides and other organisms that generate gas or toxins, offering symptomatic relief for some patients. Its long-term utility, however, is constrained by side effects and the potential for resistance or secondary fungal overgrowth, which is why it is typically used as an adjunct or for brief induction rather than maintenance (73).

More refined approaches are under active investigation. One direction focuses on removing adherent-invasive E. coli (AIEC)—a pathobiont repeatedly isolated from the ileal mucosa of Crohn’s disease patients—using specifically designed antibiotics or bacteriophage preparations. Another line of work involves encapsulating antibiotics in polymeric microspheres or related carriers to release the drug precisely at inflamed ileal or colonic segments. Such strategies aim to reduce collateral damage to the broader microbiota (74). Overall, antimicrobial strategies are shifting from broad, non-discriminatory pressure toward selective modulation that restrains pathogenic taxa while preserving commensals and mucosal immune balance. **Table 3** summarizes this paradigm shift by comparing major antibiotic classes with respect to antimicrobial spectrum, microbiota disruption, AMP-related immune effects, and long-term ecological reversibility.

**Table 3:** Ecological and immunological impacts of major antibiotic classes on the gut microbiota and antimicrobial peptide system in inflammatory bowel disease.

Antibiotic class/exemplar	Antimicrobial spectrum & exposure characteristics	Gut microbiota effects	Immunological/AMP-related effects	Ecological reversibility/long-term impact	Reference
Broad-spectrum systemic antibiotics (e.g., third-/fourth-gen cephalosporins, piperacillin–tazobactam, carbapenems, broad-spectrum fluoroquinolones, vancomycin)	High systemic exposure; broad activity; high intraluminal concentration via biliary/intestine excretion.	Sharp decline in α-diversity; depletion of SCFA-producers; expansion of Proteobacteria; increased resistome; predispose to C. difficile.	Loss of tonic PRR signaling → ↓ Paneth-cell α-defensins and RegIIIγ; impaired inducible AMP responses; secondary elevation of neutrophil AMPs during inflammation.	Dysbiosis may persist months–years; repeated exposure causes cumulative ecological imprinting; linked to increased IBD onset/relapse risk.	(59, 142, 143)	
Nitroimidazoles (e.g., metronidazole)	Anaerobe-targeted; high luminal concentration.	Suppression of anaerobic commensals (Bacteroides); expansion of Enterobacteriaceae; may increase fungal load.	↓ SCFA → ↓ IL-22/STAT3 → ↓ baseline AMP; Enterobacteriaceae expansion promotes stress-induced RegIIIβ/γ.	Short courses reversible; long-term or repeated use → persistent disruption.	(57, 61, 144, 145)	
Fluoroquinolones (e.g., ciprofloxacin, levofloxacin)	Targets Gram-negative aerobes and some Gram-positive; high oral bioavailability.	Reduced richness; selection for quinolone-resistant strains; ecological drift; fungal expansion when combined therapies.	Transient ↓ LPS-activation; resistant E. coli sustains PRR signaling and inducible AMP expression; context dependent.	Partial recovery; repeated exposure stabilizes resistant dysbiotic state.	(61, 143)	
Non-absorbed rifamycins (e.g., rifaximin)	Minimal systemic absorption (<0.5%); high gut lumen concentration.	Modest “eubiotic” shift; partial preservation of commensals; reduction of inflammatory taxa; maintains α-diversity.	Primary: epithelial PXR agonism with downstream NF-κB/PRR modulation; Secondary: modest eubiotic support of metabolite-dependent signaling.	Recovery is faster & more complete; long-term resistome impact still under study.	(68, 70, 146–148)	
Macrolides & tetracyclines (e.g., azithromycin, clarithromycin, doxycycline, minocycline)	Macrolides: Gram-positive/atypicals; tetracyclines: broad bacteriostatic; biliary elimination.	Reduced diversity; enrichment of resistant strains; altered early-life microbiota trajectories.	Protein synthesis inhibition → ↓ epithelial AMP induction; ↓ inflammatory cytokines; complex immunomodulation.	Early-life exposure → lasting shifts & increased immune-mediated disease risk.	(75, 77, 149, 150)	
Targeted or precision antimicrobials (e.g., selective suppression of pathobionts, bacteriophage-based approaches, colon-targeted delivery)	Narrow, pathobiont-focused activity with limited systemic exposure and spatially restricted effects in the gut.	Selective reduction of disease-associated taxa while largely preserving overall microbial diversity and core commensals.	Attenuates excessive PRR stimulation from specific pathobionts while maintaining commensal-driven basal antimicrobial peptide signaling.	Lower risk of durable dysbiosis and resistome expansion compared with broad-spectrum antibiotics, though long-term efficacy requires further validation.	(151–154)	

This table summarizes the spectrum of activity, microbiota remodeling effects, and antimicrobial peptide (AMP) consequences of commonly used antibiotic classes in the context of IBD. Broad-spectrum systemic antibiotics profoundly deplete commensal anaerobes, reduce baseline AMP expression, and promote a long-lasting dysbiotic state, whereas non-absorbed or targeted strategies induce more modest ecological disruption and may preserve or recalibrate AMP-mediated mucosal defense. The differential ecological footprint and reversibility of antibiotic classes emphasize the need for precision antimicrobial approaches in patients with IBD.

### 4.3 Immune-modulatory and microbiota-feedback mechanisms

Antibiotics can influence host antimicrobial peptide (AMP) expression and function through several interrelated pathways—including microbiota-mediated feedback, direct immunomodulatory effects, and longer-term ecological or resistance-driven consequences.

#### 4.3.1 Immune-modulation-driven AMP regulation mechanisms

Growing evidence suggests that several antibiotics exert immunomodulatory and anti-inflammatory actions that are not strictly dependent on their antimicrobial activity. For example, members of the tetracycline and macrolide classes have been shown to suppress neutrophil chemotaxis, reduce reactive oxygen species generation, and dampen the release of pro-inflammatory mediators—effects that, in many studies, occur independently of their direct bactericidal properties (75–77). These agents can downregulate key cytokines such as TNF-α, IL-1β, IL-6, and IL-8, and in some contexts enhance the production of anti-inflammatory cytokines including IL-10, thereby reshaping the local mucosal immune environment (78). Consistent with these observations, the pro-inflammatory cytokines and pattern-recognition receptor (PRR) pathways modulated by such antibiotics are precisely the upstream signals that drive epithelial antimicrobial peptide (AMP) expression, including β-defensin-2 (HBD-2), other β-defensins, and the cathelicidin LL-37. Experimental and translational studies have shown that the induction of HBD-2 is highly dependent on these inflammatory cues—particularly IL-1β, TNF-α, and TLR2/4-activated NF-κB and MAPK signaling cascades (79). This implies that antibiotics with immunomodulatory properties, by attenuating these pathways, could theoretically dampen the induction of AMPs at inflamed mucosal surfaces.

#### 4.3.2 Microbiota-driven AMP feedback mechanisms

Antibiotic-induced shifts in gut microbial composition can trigger compensatory adjustments in the host’s antimicrobial peptide (AMP) system. Under physiological conditions, commensal bacteria provide a steady stream of microbial signals that help maintain low, homeostatic levels of AMP production (23). When antibiotics reduce the microbial load, this tonic stimulation is lost and epithelial AMP secretion declines accordingly.

Work by Bhalodi and colleagues illustrates this feedback loop: piperacillin–tazobactam treatment depletes anaerobic commensals such as *Bifidobacterium* and *Eubacterium*, weakening basal AMP-inducing signals, whereas post-treatment rebound expansion of resistant *Enterobacteriaceae* reactivates epithelial pathways—most notably the IL-22–RegIII axis—resulting in compensatory AMP upregulation. This biphasic response reflects an initial suppression followed by overshoot of AMP output driven by antibiotic-induced dysbiosis (80).

A more pronounced compensatory response has also been observed in experimental models. In the study by Ju et al., metronidazole administration sharply reduced specific commensal taxa such as Turicibacter, while allowing Escherichia coli to expand. Strikingly, mice exposed to metronidazole showed markedly increased ileal expression of the AMPs RegIIIβ and RegIIIγ compared with untreated controls (81). These elevations likely reflect enhanced activation of innate immune pathways—potentially through IL-22—triggered by excessive E. coli growth.

Antibiotic-induced dysbiosis can elicit compensatory AMP upregulation that transiently restrains pathogenic expansion but, when sustained, may disrupt commensals and impair epithelial integrity. Thus, antibiotics function as double-edged modifiers of gut homeostasis in IBD, simultaneously shaping microbiota, immune signaling, and AMP dynamics. Recognizing this interdependence is critical for developing microbiota-sparing antibiotic strategies.

## 5 The AMP–antibiotic–microbiota triad: a dynamic cross-regulatory network in gut homeostasis

**Figure 2:**
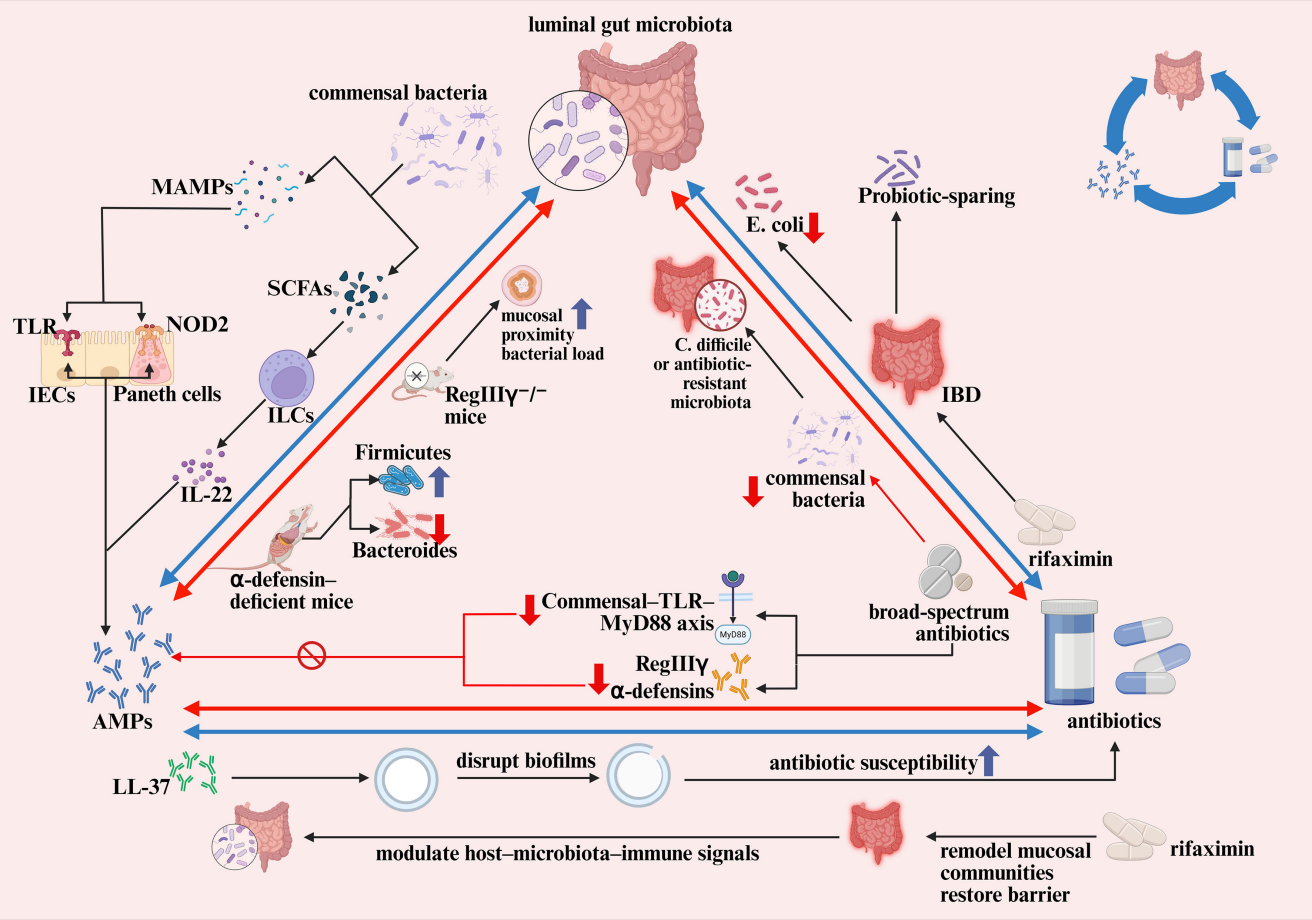
This figure illustrates the dynamic cross-regulatory relationships among antimicrobial peptides (AMPs), commensal bacteria, and antibiotics in shaping intestinal immune homeostasis. Commensal-derived molecular patterns and metabolites activate epithelial pattern-recognition pathways—including TLR, NOD2, and IL-22/STAT3 signaling—to sustain the production of α-defensins, RegIII lectins, and other AMPs that enforce microbial spatial segregation and maintain ecological balance. Genetic or functional defects in AMP secretion disrupt this equilibrium, promoting shifts in microbial composition and increased mucosal bacterial burden. Antibiotic perturbation alters these regulatory circuits by depleting commensals and weakening microbiota-dependent AMP induction, facilitating pathogen expansion and antibiotic-resistant overgrowth. In contrast, non-systemic agents such as rifaximin can preserve beneficial taxa, remodel mucosa-associated communities, and partially restore epithelial barrier function. AMPs and antibiotics also interact at the microbial interface: AMPs enhance antibiotic susceptibility through biofilm disruption, while repeated antibiotic exposure may foster resistance traits that diminish AMP effectiveness. Together, these bidirectional influences define a conditional defense triad whose balance determines whether the gut environment remains homeostatic or progresses toward dysbiosis and inflammation.

To contextualize the interactions discussed here, **Figure 2** schematically illustrates how antimicrobial peptides (AMPs), commensal microbes, and antibiotics form an integrated regulatory network. Commensal signals induce AMP production via innate and cytokine pathways, AMPs reciprocally shape microbial composition and spatial organization, and antibiotic exposure perturbs both microbial ecology and AMP-dependent mucosal defense. Together, this triad provides the dynamic basis for gut homeostasis or, when disrupted, dysbiosis and inflammation. The following subsections detail each component of this system.

### 5.1 AMP–microbiota interactions: mutualism and counterbalance

Antimicrobial peptides (AMPs) sit at the crossroads of host defense and microbial ecology, shaping the composition of the gut microbiota while being tightly regulated by microbial signals in return. This mutual interplay forms a core mechanism of intestinal homeostasis. The following sections summarize how AMPs govern microbial community structure and how commensal microbes reciprocally modulate AMP expression.

#### 5.1.1 AMPs as ecological gatekeepers of the gut microbiota

Different antimicrobial peptides (AMPs) display distinct lineage-specific activities, and consequently shape microbial diversity in highly selective ways. Among them, Paneth-cell-derived α-defensins are considered key determinants of small-intestinal microbial composition (82). In a series of elegant genetic mouse studies, Salzman and colleagues demonstrated that animals lacking mature α-defensins show a marked reduction in Bacteroidetes together with an expansion of Firmicutes, whereas transgenic mice expressing high levels of human HD5 exhibit the opposite, mirror-image shift (64). Notably, these opposing patterns occur without a change in total bacterial load, indicating that α-defensins govern which bacteria predominate rather than altering overall microbial abundance. Such compositional rewiring further influences mucosal immune responses, illustrating that AMPs help sculpt not only microbiota structure but also downstream adaptive immunity.

Beyond lineage-specific selection, AMPs maintain intestinal homeostasis through control of spatial organization of the microbiota. RegIIIγ represents a well-established example: by enforcing physical separation between microbes and the epithelial surface, it preserves a critical bacteria-free zone along the mucosa. In RegIIIγ-deficient mice, this spatial segregation collapses, resulting in close bacterial apposition to the epithelium and a substantial increase in mucosal bacterial burden (18).

Taken together, AMPs function as ecological regulators that orchestrate the distribution and competitive balance of microbes within the intestinal lumen, thereby sustaining a stable symbiotic environment. When AMP expression is compromised—whether due to genetic defects or inflammation-induced exhaustion—these ecological constraints are weakened. Bacterial groups normally kept in check may expand unchecked, while beneficial taxa decline or disappear, ultimately driving the community toward a dysbiosis and potentially pathogenic configuration (2).

#### 5.1.2 Microbial regulation of host antimicrobial peptide expression

Correspondingly, the gut microbiota also shapes host antimicrobial peptide (AMP) production through a variety of molecular cues, illustrating the reciprocal nature of host–microbe symbiosis. Resident commensals function almost like “trainers” of mucosal immunity: without provoking inflammation, they provide tonic stimulation that maintains a basal level of AMP secretion and thereby reinforces protection against invading organisms (35). Microbe-associated molecular patterns (MAMPs) provide tonic, non-inflammatory cues that help set the baseline mucosal AMP tone and maintain barrier–microbiota coexistence (83, 84). This tonic regulation depends on intact, physiologically relevant microbial signaling (35). Beyond structural MAMP cues, microbial metabolites (e.g., SCFAs and tryptophan-derived indoles) provide key inputs that calibrate AMP programs through cytokine-linked circuits discussed above (28, 34, 85).

Collectively, these interactions give rise to an elegant positive-feedback circuit: by releasing metabolites that enhance mucosal antimicrobial defenses, commensal microbes help the host eliminate competing, AMP-sensitive organisms, ultimately safeguarding the ecological niche they themselves inhabit. This mutually beneficial arrangement reflects the outcome of long-term co-evolution. For instance, certain Bacteroides species possess dedicated mechanisms that confer resistance to host AMPs, whereas competing bacteria lack such adaptations (86). As a result, Bacteroides withstand AMP-mediated pressures and outcompete sensitive strains, contributing to the stability of the microbial community.

In summary, a healthy commensal community actively tunes host AMP production, working with the mucosa to exclude pathogens and sustain intestinal ecological balance. In IBD, this finely regulated system breaks down: the loss of key commensals reduces AMP output, whereas expansion of pathobionts drives excessive AMP induction, creating a self-reinforcing cycle of dysregulated mucosal immunity.

### 5.2 Antibiotic–microbiota ecology: perturbation and counterbalance

Building upon the mechanistic framework established in Section 6, this section shifts to a systems-level perspective, examining the ecological and evolutionary consequences of antibiotic perturbation within the AMP–microbiota network. Antibiotics introduce major disturbances into the gut ecosystem, rapidly altering microbial composition and host immune dynamics. Yet these effects are not unidirectional—the microbiota also adapts to repeated antibiotic pressure, reshaping its resistance traits and ecological behavior. The following sections outline both sides of this perturbation–counteradaptation cycle and their consequences for AMP regulation and mucosal stability.

#### 5.2.1 Disruptive forces: antibiotics within the AMP–microbiota–host triad

Antibiotic exposure acts as an exogenous perturbation that shifts the triad network toward alternative ecological configurations (59). Such shifts alter the relational balance among microbial composition, resistance traits, and host defense. Selective depletion generates ecological vacancies that reshape competitive hierarchies within the community (87). Sensing these compositional changes through pattern-recognition pathways and microbially derived metabolites, the host correspondingly adjusts AMP expression to re-establish mucosal equilibrium (13).

Clinically, antibiotic-associated colitis provides a clear illustration of this sequence: broad-spectrum antibiotics eradicate large portions of the resident microbiota, allowing a few resistant organisms—most notably Clostridioides difficile—to expand unchecked, triggering severe inflammation as the community architecture collapses (88). At this point, innate defenses alone are usually insufficient to eliminate the pathogen, making targeted antimicrobial therapy or fecal microbiota transplantation necessary to restore ecological balance (89). Taken together, antibiotics reshape not only the microbial ecosystem but also exert cascading effects on mucosal immunity, positioning them as one of the most disruptive forces within this three-way interaction system.

#### 5.2.2 Bidirectional adaptation in the antibiotic–microbiota relationship

On the other hand, the gut microbiota as a whole also adapts to repeated antibiotic exposure, developing collective feedback responses over time. At the community level, frequent courses of antibiotics impose strong selective pressures that allow resistant strains to dominate, thereby diminishing the efficacy of subsequent treatments (90). The spread of such resistance further compromises the action of host antimicrobial peptides (AMPs), as many bacterial defense strategies—such as reduced membrane permeability and high-capacity efflux pumps—protect against both antibiotics and AMPs, creating well-recognized patterns of cross-resistance (91, 92).

In addition, repeated antibiotic-induced stress favors the enrichment of metabolically quiescent, persisted-like subpopulations. These low-activity cells are intrinsically tolerant to bactericidal antibiotics and can persist long after therapy ends (93). Accumulating evidence indicates that persister cells serve as a nidus for chronic or recurrent infections and may function as ongoing microbial stimuli in chronic inflammatory conditions such as IBD, sustaining mucosal immune activation (94, 95).

Notably, the gut microbiota does not invariably return to its pre-treatment configuration once antibiotics are withdrawn. Certain commensal taxa that are highly sensitive to antibiotics may remain at very low abundance—or be lost altogether—creating persistent ecological voids. In such cases, the restoration of their associated functions may require exogenous ecological reconstruction, including targeted probiotic supplementation or fecal microbiota transplantation (96).

Taken together, these observations highlight a form of mutual conditioning between antibiotics and the gut microbiota: antibiotics reshape community structure, while the microbiota, through evolutionary and ecological adjustments, modifies its collective responsiveness to antibiotic pressure (90). Throughout this dynamic process, the host AMP system is also brought into play, attempting to compensate for or complement antibiotic activity at different stages of microbial disturbance and recovery.

### 5.3 AMPs and antibiotics: synergy, conflict, and clinical implications

The interaction between AMPs and antibiotics is not unidimensional; it spans a spectrum from therapeutic synergy to functional conflict, and recognizing this duality is essential for optimizing anti-infective strategies and preserving mucosal immune balance.

#### 5.3.1 Synergistic antimicrobial actions of AMPs and antibiotics

In an ideal scenario, antimicrobial peptides (AMPs) and antibiotics act in concert toward a common target, each fulfilling its role in suppressing excessive pathogen proliferation. Multiple studies have demonstrated that AMPs such as LL-37 can disrupt biofilm architecture and enhance bacterial susceptibility to antibiotics. Pletzer et al. further reported that antibiofilm peptides promote biofilm disassembly, thereby increasing bacterial exposure to antibiotics and producing a synergistic antimicrobial effect (97). Antibiotics, by reducing the overall bacterial burden, may indirectly augment the defensive efficacy of endogenous AMPs (98).

Evidence suggests that during the late stages of infection or under low-density residual bacterial conditions, AMPs exhibit enhanced bactericidal activity, characterized by sustained disruption of membrane permeability and metabolic function (99). This dual-action mechanism—one targeting external microbial membranes and the other mediated through host innate defenses—may help explain the clinical success observed in certain infection-related complications of inflammatory bowel disease (IBD). For instance, postoperative administration of nitroimidazole antibiotics in Crohn’s disease patients has been shown to reduce recurrence rates (100).

Animal studies indicate that rifaximin can remodel mucosa-associated microbial communities, alleviate mucosal inflammation or dysfunction, and restore epithelial barrier integrity by suppressing pro-inflammatory signaling pathways such as TLR4/NF-κB and by modulating host–microbiota–immune interactions (67, 101). In recent years, the concept of “combined antimicrobial therapy”—using exogenous AMP-like molecules together with conventional antibiotics—has gained attention as a promising approach for treating refractory infections (102). Preclinical models have demonstrated that certain AMP–antibiotic combinations exert synergistic bactericidal effects, enabling dose reduction and broadening of the antibacterial spectrum (103).

Within the context of IBD, this therapeutic rationale may also hold promise: supplementing patients with deficient AMPs (e.g., defensin or lysozyme mimetics) while minimizing broad-spectrum antibiotic exposure could help restore microbial homeostasis and reinforce mucosal immunity.

#### 5.3.2 The AMP–antibiotic mismatch: microbiota disruption and immune consequences

Although antimicrobial peptides (AMPs) and antibiotics are traditionally regarded as complementary arms of infection control, accumulating evidence indicates that antibiotic exposure may functionally conflict with endogenous AMP-mediated defense. This tension extends beyond immediate antimicrobial interactions and reflects deeper evolutionary constraints. At an evolutionary level, an additional layer of conflict emerges: AMP systems coevolved with host-associated microbial communities as nuanced and homeostasis-oriented defense strategies, whereas modern antibiotics represent exogenous interventions that may not be aligned with this coadapted axis (104). Such “evolutionary asynchrony” has been proposed to disrupt innate recognition of microbial cues. Given that AMP systems evolved under conditions of persistent, low-grade microbial stimulation, the abrupt withdrawal of these signals through antibiotic exposure may recalibrate baseline immune regulation and increase susceptibility to disproportionate inflammatory responses to otherwise harmless antigens (104–106).

Viewed from microecological, immunological, and evolutionary perspectives, antibiotic-mediated disruption of the AMP–microbiota axis provides a unifying framework for antibiotic-associated inflammatory phenotypes. Together, antimicrobial peptides, antibiotics, and the gut microbiota form a dynamic, context-dependent defense network: preserved AMP-mediated control and judicious antibiotic use support mucosal homeostasis, whereas AMP insufficiency, repeated antibiotic exposure, and persistent dysbiosis reinforce impaired host defense and chronic inflammation in IBD. This network-based view shifts therapy from isolated targets toward recalibrating antimicrobial balance by strengthening AMP function, minimizing disruptive antibiotic effects, and restoring microbial resilience. The following section outlines integrative strategies to re-establish balance within the AMP–antibiotic–microbiota triad.

## 6 Rebuilding antimicrobial balance: network-level strategies targeting the AMP–antibiotic–microbiota axis

Inflammatory bowel disease reflects a breakdown of antimicrobial balance, driven by impaired AMP production, antibiotic-induced ecological disruption, and pathogenic expansion. Restoring mucosal homeostasis therefore requires coordinated realignment of the AMP–antibiotic–microbiota axis rather than isolated anti-inflammatory intervention.

### 6.1 AMP:restore innate defense

Restoring antimicrobial peptide (AMP) function is increasingly regarded as an important strategy in re-establishing host–microbiota defensive balance. Key regulatory pathways—including the vitamin D–VDR axis, short-chain fatty acids, and IL-22–STAT3 signaling—have been shown to induce epithelial production of defensins, cathelicidin (LL-37), and RegIII lectins, thereby strengthening intrinsic mucosal antimicrobial capacity (107–109). Several synthetic AMPs, such as OP-145, have demonstrated potent antibacterial and antibiofilm activity against multidrug-resistant clinical isolates *in vitro*, suggesting their potential as adjuncts or alternatives to conventional antibiotics. Likewise, lactoferrin-derived peptides exhibit broad-spectrum antimicrobial activity against bacterial, fungal, and parasitic pathogens (110, 111). However, whether such molecules can restore intestinal barrier integrity or selectively reconfigure gut microbial ecology remains to be determined in relevant *in vivo* models (112).

An additional consideration is the potential for partial cross-resistance between antibiotic-induced bacterial adaptations and host-directed AMP therapeutics (113, 114). Importantly, such adaptations do not uniformly preclude AMP-based therapy but instead highlight heterogeneity in bacterial adaptive states following antibiotic exposure (115). The efficacy of AMP mimetics may therefore depend on resistance mechanisms present within a given microbial community (104). These considerations underscore the importance of precision deployment strategies—integrating prior antibiotic exposure history, resistome profiling, and microbial functional assessment—to optimize the therapeutic potential of AMP-based intervention.

### 6.2 Antibiotics:reduce ecological disruption

Secondly, the paradigm of antibiotic use is shifting from indiscriminate eradication toward controlled and microbiota-conscious modulation, emerging as a key strategy in restoring balance within the AMP–antibiotic–microbiota triad. In pathogen-driven subtypes of IBD, targeted antimicrobial interventions—such as therapies directed against AIEC, Fusobacterium, or Mycobacterium avium subspecies paratuberculosis—have reported trends toward improved inflammatory control and mucosal healing in selected studies, suggesting that precision antimicrobial approaches may impose less collateral disruption to commensal ecosystems than traditional broad-spectrum regimens (116–118). Nevertheless, the reproducibility, patient selection, and long-term safety of such strategies remain to be fully established in prospective trials. In parallel, advances in antibiotic delivery have enabled precision to extend beyond the choice of agent to its spatial and temporal deployment, exemplified by colon-targeted release systems designed to suppress pathogenic taxa while minimizing off-target effects on symbiotic microbes and potentially creating a therapeutic window for re-colonization and ecological restoration (119).

### 6.3 Microbiota:rebuild symbiotic architecture

Finally, microbiota replacement and transplantation constitute the ecological pillar within this tripartite framework. Fecal microbiota transplantation (FMT), as the most established modality, has achieved notable rates of clinical remission in several studies, including randomized trials in patients with moderate ulcerative colitis, supporting the concept that restoring microbial diversity and function may confer therapeutic benefit in IBD (120). Systematic evaluations further indicate that donor microbiomes characterized by higher alpha-diversity, an abundance of short-chain fatty acid (SCFA)–producing taxa, and enrichment of Clostridium clusters IV/XIVa are more frequently associated with favorable outcomes, suggesting that these microbial features may inform donor selection strategies (121). Increasing experimental evidence has shown that SCFAs can activate IL-22/STAT3 signaling and stimulate epithelial antimicrobial peptide (AMP) production, thereby reinforcing mucosal barrier defense (107). This raises the possibility that restoring short-chain fatty acid (SCFA)–producing capacity via microbiota transplantation may synergize with endogenous AMP-mediated immunity to enhance epithelial stability and ecological recovery, although the durability and clinical relevance of this pathway remain to be established. Given the limitations of conventional fecal microbiota transplantation (FMT), including variability, standardization challenges, and safety concerns, next-generation microbial therapeutics—such as engineered probiotic consortia, defined microbial communities, and synthetic symbiotic ecosystems—are emerging as more controllable strategies for sustaining long-term gut homeostasis (120, 122). Nevertheless, these ecological interventions require large-scale, longitudinal studies to clarify their efficacy, safety profiles, and capacity to achieve stable engraftment.

IBD management is moving toward an ecological paradigm focused on restoring balance within the AMP–antibiotic–microbiota triad. Strengthening epithelial AMP defenses, minimizing antibiotic-induced disruption, and rebuilding symbiotic microbial ecosystems may act synergistically to re-establish mucosal homeostasis and enable more durable, personalized disease control.

## 7 Conclusions and future directions

Inflammatory bowel disease exemplifies the consequences of disrupted coordination between antimicrobial defenses and the intestinal microbiota. As highlighted throughout this review, endogenous antimicrobial peptides, antibiotic pressures, and the gut microbiota constitute an interdependent regulatory network rather than isolated components of host defense. Conceptualizing these elements as an integrated AMP–antibiotic–microbiota triad provides a mechanistic framework for understanding how endogenous and exogenous antimicrobial forces converge to shape mucosal inflammation. When balanced, AMPs constrain bacterial encroachment and support epithelial integrity, while judicious antibiotic use limits pathogenic expansion without destabilizing commensal communities. Disruption of this equilibrium—through AMP insufficiency, altered microbial resilience, or excessive antimicrobial exposure—amplifies dysbiosis, compromises barrier function, and sustains chronic intestinal inflammation.

Future advances will require a shift from descriptive models toward predictive frameworks that integrate microbial, epithelial, and pharmacologic perturbations across mucosal ecosystems. Defining the context-dependent functions of AMPs and delineating how distinct antibiotic classes modulate AMP pathways and microbial recovery will be essential for identifying interventions that preserve commensal-driven protection while avoiding immune destabilization.

Emerging therapeutic strategies increasingly reflect this ecological perspective, including microbiota-sparing antibiotics, AMP-enhancing approaches, targeted microbial metabolites, and multi-omics–guided patient stratification. Ultimately, durable remission in IBD may depend on realigning endogenous and exogenous antimicrobial forces to reinforce both host defense and microbial coexistence. By positioning antimicrobial balance as a dynamic, triadic regulatory system rather than a linear pathway, this framework may inform more precise and sustainable therapeutic strategies and clarify shared mechanisms across immune-mediated diseases driven by disrupted host–microbe interactions.
